# Peer Phubbing and Social Networking Site Addiction: The Mediating Role of Social Anxiety and the Moderating Role of Family Financial Difficulty

**DOI:** 10.3389/fpsyg.2021.670065

**Published:** 2021-08-05

**Authors:** Xiaoyuan Chu, Shutian Ji, Xingchao Wang, Jingyue Yu, Yuxin Chen, Li Lei

**Affiliations:** ^1^School of Economics and Management, Beijing University of Posts and Telecommunications, Beijing, China; ^2^Research Center of Development Strategy for the High-level Featured University, Beijing University of Posts and Telecommunications, Beijing, China; ^3^Department of Psychology, Faculty of Science and Technology, Renmin University of China, Beijing, China; ^4^School of Educational Science, Shanxi University, Taiyuan, China; ^5^School of Sociology and Population Studies, Faculty of Social Sciences, Renmin University of China, Beijing, China; ^6^School of Education, Faculty of Social Sciences, Renmin University of China, Beijing, China

**Keywords:** peer phubbing, social networking site addiction, social anxiety, family financial difficulty, undergraduate

## Abstract

A growing body of research has pinpointed the consequences as well as mechanisms of phubbing. However, few studies have explored the relationship between peer phubbing and social networking site addiction. Based on the self-determination theory, the exclusion theory of anxiety, the social compensation model, and the reserve capacity model, the present study examined whether peer phubbing was positively related to social networking site addiction among undergraduates, whether social anxiety mediated the relationship, and whether this mediating process was moderated by family financial difficulty. Our theoretical model was tested using the data collected from 1,401 Chinese undergraduates (*M*_age_ = 18.83 years, standard deviation = 0.93). The participants completed anonymous questionnaires that assessed their peer phubbing, social anxiety, social networking site addiction, and family financial difficulty. The correlation analysis indicated that peer phubbing was positively associated with social networking site addiction. The testing for moderated mediation further revealed that social anxiety partially mediated the association between peer phubbing and social networking site addiction, with family financial difficulty moderating the first stage. To be specific, the indirect association between peer phubbing and social networking site addiction *via* social anxiety was stronger for undergraduates in high family financial difficulty. The results from this study extend research on the potential consequences of phubbing as well as highlight the significance of uncovering the underlying mechanisms.

## Introduction

Due to its constantly enhancing functions, ownership and use of a mobile phone have skyrocketed in recently years. In March 2020, 896.90 million Chinese accessed the Internet using mobile phones (China Internet Network Information Center (CNNIC), 2020). In addition to tremendous practical and social benefits of mobile phones, some emerging problems can be observed. “Phubbing” is one among them. This term, a portmanteau of “phone” and “snubbing,” describes the act of ignoring other people in the context of social contact by paying attention to his/her phone instead of focusing on the person directly in his/her company (Vanden Abeele et al., 2019; Bai et al., 2020; Ivanova et al., 2020). It is deemed as a kind of social exclusion and interpersonal neglect (Xie et al., 2019), which often disturbs the ongoing interpersonal communication (Hong et al., 2019; Liu et al., 2019). This behavior is usually perceived as disrespectful and socially inappropriate (Vanden Abeele et al., 2016) but still can be observed continuously in various demographic groups (Karadaǧ et al., 2015; Liu et al., 2019). A phubber and phubbee are the two parties involved in the phubbing behavior. The phubber is the person who starts phubbing the interlocutor, and the phubbee is the recipient of the phubbing behavior (Chotpitayasunondh and Douglas, 2018a). The potential consequences have been identified for the phubbees. Being phubbed threats the need for belongingness (Chotpitayasunondh and Douglas, 2018a) and is positively associated with anxiety, depression, somatization, and hostility (Ergün et al., 2019). Diverse dyads might exhibit different problems. For example, parental phubbing not only reduces the mental health of adolescents (Bai et al., 2020) but also increases their academic burnout (Bai et al., 2020), depression (Xie and Xie, 2019; Wang et al., 2020), and problematic use of mobile phones (Hong et al., 2019). Partner phubbing undermines the relationship satisfaction and ultimately the personal well-being (Roberts and David, 2016; Wang et al., 2017). Boss phubbing reduces supervisory trust, engagement, psychological meaningfulness, psychological availability, job satisfaction, and performance of the employee (Roberts and David, 2017, 2020).

Few studies have explored the phubbing behavior among undergraduates. They are the most rapid adopters of mobile phone technology (Lepp et al., 2014), among whom the ownership of mobile phones is universal (Long et al., 2016; Zhang et al., 2020). Besides, being ostracized occurs regularly for the young adults (Nezlek et al., 2012) with a serial of behavioral and psychological consequences (Bacon and Engerman, 2018). The primary interpersonal connections for undergraduates are connections to family, peers, and academic institutions (Ploskonka and Servaty-Seib, 2015). Many students would launch from their families and relocate to other cities for pursuing higher education in China (Pan, 2017). Hence, along with the decreasing social connection with their families (Bacon and Engerman, 2018), their relationship with peer group become particularly important (Duncan et al., 2018). According to the above literature, it would be of great value to explore the potential consequences of peer phubbing for undergraduates.

### Peer Phubbing and Social Networking Site Addiction

According to the self-determination theory (Deci and Ryan, 2000), the need for relatedness is one of the innate psychological needs. Individuals actively pursue the relationship to satisfy the need. In non-supportive situations, where the need for relatedness cannot be well-met, substitute motives, non-autonomous regulatory styles, and rigid behavior patterns might be developed to preserve as much need satisfaction as seems possible (Deci and Ryan, 2000). Phubbing is a kind of social exclusion and interpersonal neglect (Xie et al., 2019), which threats the need for relatedness and drives the phubbee to find other ways to connect to others. The online social networking is an ideal option for the phubbees because it is secure (Valkenburg and Peter, 2009; Chen et al., 2019; Lin et al., 2019) and convenient, where they can access whenever they want to and they can interact with people living thousands of miles away. The need for relatedness can be satisfied in this way, along with social networking site addiction as a byproduct (Kardefelt-Winther, 2014). In line with this notion, prior studies have found that being phubbed by parents is positively related to mobile phone addiction for children and adolescents (Hong et al., 2019; Liu et al., 2019; Xie et al., 2019). Peer phubbing may also be a drive for social networking site addiction among undergraduates. An earlier study has indicated that peers play a significant role in the satisfaction of the need for relatedness of undergraduates (Basson and Rothmann, 2018). The ostracism experience at school settings can be positively associated with Internet addiction for University students (Poon, 2018). An experimental study also has shown that being phubbed can result in an attachment to social media (David and Roberts, 2017). Based on the above literature, it is reasonable to deduce the following hypothesis:

Hypothesis 1: Peer phubbing will be positively related to social networking site addiction for undergraduates.

### The Mediating Role of Social Anxiety

Social anxiety can be another potential consequence of peer phubbing for undergraduates. According to the exclusion theory of anxiety (Baumeister and Tice, 1990), derived from the need to belong, anxiety arises in response to an actual or threatened exclusion from important social relationships. When encountering real or perceived breaking of social connections, individuals would see themselves as incompetent, guilty, or unattractive, with social anxiety as one of the further consequences (Baumeister and Tice, 1990). In line with this notion, an empirical study has demonstrated that ostracism distress induced by social exclusion is positively correlated with anxiety (Davidson et al., 2019). Phubbing is a kind of social exclusion (Xie et al., 2019), in which the phubber neglects or ignores the phubbee with attention to the mobile phone use (Xie and Xie, 2019). A prior study also has indicated that being phubbed is positively correlated with anxiety (Ergün et al., 2019). Despite the scarce empirical evidence, it is logical to deduce that peer phubbing can be positively associated with social anxiety.

Social anxiety can be a potential proximal cause for social networking site addiction. As the social compensation model posits, individuals with less social support or interaction would use online social networking to connect with others (Kraut et al., 2002). Individuals who are socially anxious usually have difficulty to meet the need for social interaction in the real life (Chen et al., 2019). They can compensate the unsatisfying interpersonal connections in the real life by establishing relationships with the application of online social networking (Valkenburg and Peter, 2009). There, they can stay anonymous, hide physical signs of anxiety, and have better sense of control (Stritzke et al., 2004; Tian, 2013; Darcin et al., 2016), all of which reduce the fear of being evaluated negatively (Valkenburg and Peter, 2009). Although their needs for social interaction can be met in the online settings along with improved emotions, they may become addicted to the online social networking (Kardefelt-Winther, 2014). In line with this notion, social anxiety is found to be positively correlated with social networking site addiction repeatedly by the empirical studies (Chen et al., 2019; Lin et al., 2019). A meta-analysis has shown that the social anxiety is positively linked to feelings of comfort online and Internet addiction (Prizant-Passal et al., 2016). Moreover, a longitudinal study has confirmed that anxiety is an important predictor for Internet addiction (Li et al., 2019). Based on the above literature, we put forward the following hypothesis:

Hypothesis 2. Social anxiety will mediate the association between peer phubbing and social networking site addiction.

### The Moderating Role of Family Financial Difficulty

The economical and interpersonal factors can jointly influence the health of a student. According to the reserve capacity model (Gallo and Matthews, 2003), the stressful environment of poor people reduces their reserve capacity to manage a negative stimulus, with increased vulnerability to negative emotions and cognitions. In other words, individuals in high family financial difficulty have a lower capacity to deal with negative social interactions (Gallo and Matthews, 2003). Although negative social interaction (i.e., ostracism and phubbing) is a risk factor in general (Davidson et al., 2019; Xie et al., 2019), individuals under high economic stress might consider it more serious: compared with their rich counterparts, they are more vigilant (Kraus et al., 2011) and more reactive (Kraus et al., 2012) toward social threats. Their physical reactivity is stronger when encountering social threats (Chen and Matthews, 2001). Besides, they not only inclined to develop social anxiety (Cheng et al., 2015) but also tend to score high for excessive Internet use (Urbanova et al., 2019) and compulsive use of social networking sites (Cock et al., 2014). Hence, it is reasonable to deduce the following hypothesis:

Hypothesis 3. Undergraduates under higher family financial difficulty will be more reactive to peer phubbing. Family financial difficulty may moderate two links of the associations between peer phubbing and social networking site addiction: the direct link between peer phubbing and social networking site addiction and the link between peer phubbing and social anxiety.

### The Present Study

**Figure 1:**
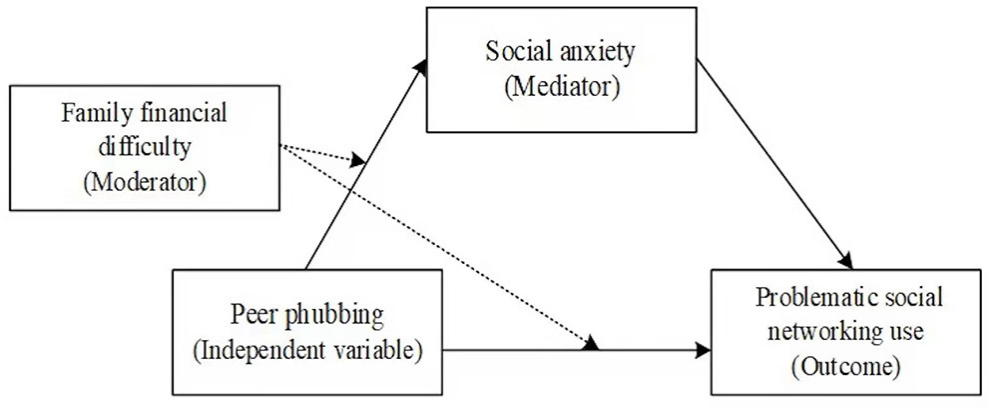
The proposed moderated mediation model.

The consequences of phubbing have gained increasing academic attention in recent years, and some underlying mechanisms have been uncovered (Chotpitayasunondh and Douglas, 2018a; Ergün et al., 2019; Xie and Xie, 2019; Bai et al., 2020; Wang et al., 2020). But much is still unknown for this relatively new phenomenon. Yet, few studies have taken the behavior of peers into consideration even though the relatedness satisfaction from his/her peer group is an important factor for mental health of undergraduates (Duncan et al., 2018). The present research aimed to fill this gap by testing whether peer phubbing is associated with social networking site addiction and identifying the underlying mechanism. A process-oriented approach was taken to test a moderated mediation model (see Figure 1) of peer phubbing and social networking site addiction. In particular, we aimed to uncover three research questions, namely, (a) to test whether peer phubbing positively associates with social networking site addiction; (b) to examine whether peer phubbing indirectly relates to social networking site addiction through social anxiety; and (c) to confirm whether the indirect relationship between peer phubbing and social networking site addiction would be moderated by family financial difficulty.

## Materials and Methods

### Participants

This survey was conducted based on a pencil-to-paper format from October to November, 2019, at two universities in Beijing, China. The initial sample comprised of 1,565 undergraduates with the response rate of 92.06%; however, after data cleansing was administered (for details, see the section “Statistical analysis”), the final sample size was reduced to 1,401 (582 females; 819 males). After obtaining the informed consent, the participants completed questionnaires including demographics, peer phubbing, social anxiety, social networking site addiction, and family financial difficulty. The average age of the sample was 18.83 years (standard deviation [*SD*] = 0.93, ranging from 16 to 23).

### Procedure

The materials and procedures of the current study were approved by the Human Research Committee of the University of the author prior to recruitment. The data were collected in the classroom of the two universities during October to November, 2019. After obtaining the informed consent, research assistants who were trained conducted the survey with standardized instructions. The anonymity was ensured, and the participants could withdraw from the study at any time with no negative consequences.

### Measures

#### Peer Phubbing

The Generic Scale of Being Phubbed (Chotpitayasunondh and Douglas, 2018b) was adapted to assess the extent to which undergraduates perceive experiencing peer phubbing. The scale consists of 22 items with three factors, namely, perceived norms, feeling ignored, and interpersonal conflict (Chotpitayasunondh and Douglas, 2018b). The only change to each item is the replacement of the word “others” with “my friends/classmates.” Participants rated each item on a 7-point scale ranging from 1 (i.e., never) to 7 (i.e., always). The responses across the 22 items were summed, with the total scores ranging from 15 to 105. A higher score represents a higher frequency of behaviors of peer phubbing. An example item is “My friends/classmates pay attention to their phones rather than talking to me.” In the confirmatory factor analysis (CFA), the results showed that the structural validity of the scale was acceptable, with standardized root mean square residual (SRMR) = 0.06, comparative fit index (CFI) = 0.91, and Tucker–Lewis index (TLI) = 0.89. The scale also had solid internal consistency (Cronbach's α = 0.96).

#### Social Anxiety

Social anxiety was assessed by the Social Interaction Anxiety Scale developed by Mattick and Clarke (1998) and revised by Fergus et al. (2012). The scale contains six items capturing the fears of general social interaction. An example item is “Nervous mixing with people when don't know well.” Items were responded on a 5-point scale ranging from 0 (i.e., not at all) to 4 (i.e., extremely). The responses were summed with total scores varying between 0 and 24. The higher scores indicate a stronger social anxiety. In the current study, CFA suggested that the structural validity of the scale was acceptable with SRMR = 0.05, CFI = 0.91, and TLI = 0.86. The Cronbach's α was 0.82.

#### Social Networking Site Addiction

Social networking site addiction was measured by the Social Networking Sites Addictive Tendencies Scale, which was developed by Milošević-Dordević and ŽeŽelj (2014). The participants read six items and reported their level of agreement with statements related to the interference with vital life activities and domination of virtual over real interpersonal relationships on a 5-point scale ranging from 1 (i.e., completely disagree) to 5 (i.e., completely agree). An example item is “Sometimes I have an impression that I live two lives: one private and another virtual.” The responses were summed with total scores varying between 5 and 30. A higher score represents a higher tendency of social networking site addiction. For the current study, CFA suggested that the structural validity of the scale was acceptable with SRMR = 0.05, CFI = 0.91, and TLI = 0.85. The measure also demonstrated a good reliability (Cronbach's α = 0.75).

#### Family Financial Difficulty

Family financial difficulty was measured by the economic strain items of the Responses to Stress Questionnaire (Wadsworth and Compas, 2002), revised by Wang et al. (2010). Specifically, the participants indicated the frequency of experiencing family financial difficulty on a 5-point scale ranging from 1 (i.e., not at all) to 5 (i.e., always). An example item is “We didn't have enough money for new clothes.” The responses of the four items were averaged with higher scores representing more family financial difficulty. In the present study, the CFA model generated a good fit, with SRMR = 0.03, CFI = 0.97, TLI = 0.90. Cronbach's α was 0.87.

### Statistical Analysis

The data analysis was conducted using the SPSS 25.0 software. Before statistical analysis, responses with the missing data were excluded from the data processing since the proportions of missing data for all variables were low (<2%). Then, the descriptive statistics and bivariate correlations were calculated for each variable. Later, Hayes' PROCESS macro Model 4 (Hayes, 2013) was performed to test the mediating role of social anxiety in the association between peer phubbing and social networking site addiction. Finally, whether the mediation process was moderated by family financial difficulty was further explored with Hayes' PROCESS macro Model 8 (Hayes, 2013). The gender and age were controlled during the analysis of models 4 and 8. Before the application of models 4 and 8, all variables were standardized except for dummy variable (i.e., gender, which was coded 0 for females and 1 for males), and the interaction terms were computed from the standardized variables. The bias-corrected percentile bootstrap method (with 5,000 resamples) was applied to determine whether the effects were significant.

## Results

### Testing for Common Method Bias

To test the common method bias, the one-factor model test was performed. The results did not provide an adequate fit to the data: SRMR = 0.12, CFI = 0.59, and TLI = 0.56. Hence, the common method bias was not considered a serious problem for this study.

### Preliminary Analysis

The descriptive statistics and correlation matrix (zero-order Pearson's correlation coefficients) of peer phubbing, social networking site addiction, social anxiety, and family financial difficulty are presented in Table 1. As expected, peer phubbing was positively associated with social networking site addiction (*r* = 0.29, *p* < 0.001) and social anxiety (*r* = 0.28, *p* < 0.001). In addition, social anxiety was positively associated with social networking site addiction (*r* = 0.39, *p* < 0.001). Moreover, family financial difficulty was positively associated with peer phubbing (*r* = 0.19, *p* < 0.001), social networking site addiction (*r* = 0.24, *p* < 0.001), and social anxiety (*r* = 0.19, *p* < 0.001). Given that peer phubbing was positively correlated to social networking site addiction, Hypothesis 1 was supported.

**Table 1:** Descriptive statistics and correlations among variables.

		***M***	***SD***	**1**	**2**	**3**	**4**
1.	PP	59.60	22.77	1			
2.	SNSA	14.21	4.60	0.29***	1		
3.	SA	7.15	4.56	0.28***	0.39***	1	
4.	FFD	1.91	0.97	0.19***	0.24***	0.19***	1

*N = 1,401; PP, peer phubbing; SNSA, social networking site addiction; SA, social anxiety; FFD, family financial difficulty*.

*** *p < 0.001*.

### Testing for the Mediating Role of Social Anxiety

In accordance with the anticipation of Hypothesis 2, social anxiety played a mediating role in the relationship between peer phubbing and social networking site addiction. Model 4 of PROCESS macro (Hayes, 2013) was performed to test this hypothesis. The result indicated that peer phubbing was positively associated with social anxiety (*b* = 0.27, *p* < 0.001, see Model 2 of Table 2), which in turn was positively related to social networking site addiction (*b* = 0.34, *p* < 0.001, see Model 3 of Table 2). Meanwhile, the direct effect of peer phubbing on social networking site addiction was also significant (*b* = 0.20, *p* < 0.001, see Model 3 of Table 2). The bias-corrected percentile bootstrap result revealed that the indirect effect of peer phubbing on social networking site addiction *via* social anxiety was significant (indirect effect = 0.09, standard error [*SE*] = 0.01, 95% confidence interval [*CI*] = [0.07, 0.12]), which accounted for 31.47% of the total effect. Therefore, social anxiety partially mediated the association between peer phubbing and social networking site addiction. This result supported Hypothesis 2.

**Table 2:** Testing the mediation effect of peer phubbing on social networking site addiction *via* social anxiety.

**Predictor**	**Model 1: SNSA**		**Model 2: SA**		**Model 3: SNSA**	
	***b***	***t***	***b***	***t***	***b***	***t***
PP	0.29	11.40***	0.27	10.57***	0.20	7.99***
SA					0.34	13.49***
Gender	0.03	0.52	−0.07	−1.25	0.05	1.00
Age	−0.03	−1.21	0.04	1.53	−0.04	−1.84
*R^2^*	0.09		0.08		0.19	
*F*	43.34***		40.89***		82.23***	

*N = 1,401. Each column is a regression model that predicts the criterion at the top of the column. SNSA, social networking site addiction; PP, peer phubbing; SA, social anxiety*.

*** *p < 0.001*.

### Testing for Moderated Mediation

**Figure 2:**
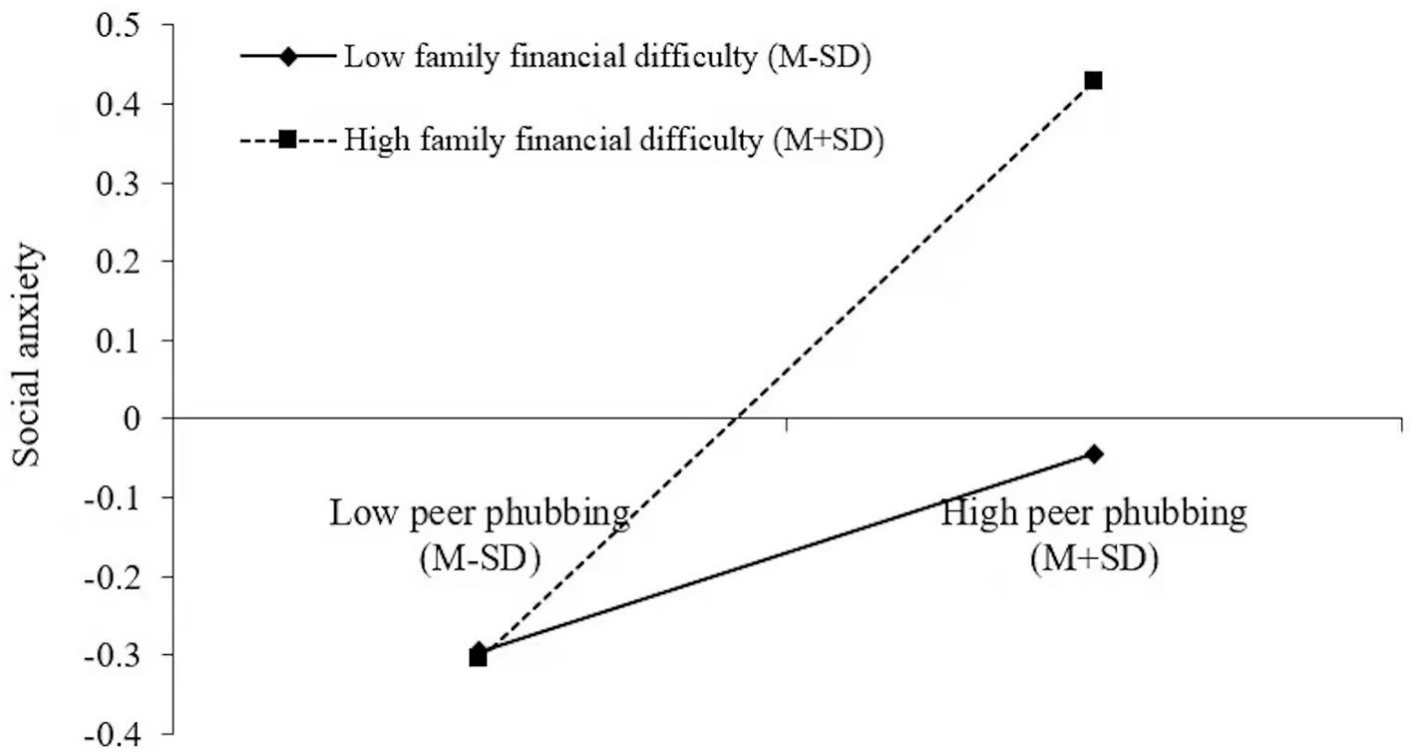
Family financial difficulty moderates the relationship between peer phubbing and social anxiety.

In Hypothesis 3, the family financial difficulty was anticipated to moderate the indirect relationships between peer phubbing and social networking site addiction *via* social anxiety. To examine the hypothesis, Model 8 of PROCESS macro (Hayes, 2013) was performed. The result indicated that the first stage of the indirect effect was moderated by family financial difficulty (*b* = 0.12, *p* < 0.001, see Model 1 of Table 3). For clarity, we plotted peer phubbing on social anxiety (Figure 2), separately for participants with low and high family financial difficulty (1 *SD* below the mean and 1 *SD* above the mean, respectively). The simple slope tests indicated that for undergraduates with low family financial difficulty, social anxiety had a significantly ascending trend as the increase of peer phubbing (*b*_simple_ = 0.13, *t* = 3.49, *p* < 0.001); for undergraduates in high family financial difficulty, the ascending trend of social anxiety was more obvious as the increase of peer phubbing (*b*_simple_ = 0.37, *t* = 10.18, *p* < 0.001). The indirect effect of peer phubbing on social networking site addiction *via* social anxiety was stronger for undergraduates with high family financial difficulty (indirect effect = 0.12, *SE* = 0.02, 95% *CI* = [0.09, 0.15]) than those with low family financial difficulty (indirect effect = 0.04, *SE* = 0.01, 95% *CI* = [0.02, 0.07]). The difference between the two indirect effects was significant (difference between the indirect effects = 0.07, *SE* = 0.02, 95% *CI* = [0.04, 0.11]). Therefore, Hypothesis 3 was supported.

**Table 3:** Testing the moderated mediation effect of peer phubbing on social networking site addiction.

**Predictor**	**Model 1: SA**		**Model 2: SNSA**	
	***b***	***t***	***b***	***t***
PP	0.25	9.55***	0.18	7.11***
SA			0.32	12.62***
FFD	0.12	4.42***	0.15	6.05***
PP*FFD	0.12	5.11***	−0.01	−0.53
Gender	−0.08	−1.49	0.03	0.60
Age	0.02	0.87	−0.05	−2.28*
*R^2^*	0.12		0.21	
*F*	36.53***		62.32***	

N = 1,401. Each column is a regression model that predicts the criterion at the top of the column. SNSA, social networking site addiction; PP, peer phubbing; SA, social anxiety; FFD, family financial difficulty; PP*FFD, interaction of peer phubbing and family financial difficulty.

* *p < 0.05*,

*** *p < 0.001*.

## Discussion

The consequences of being phubbed have garnered much empirical evidence (Roberts and David, 2016, 2020; Hong et al., 2019; Bai et al., 2020); however, few studies have yet explored the consequences of being phubbed by peers. Besides, few studies have focused on undergraduates. Being phubbed by peers can be a familiar phenomenon for undergraduates who are regular users of the mobile phones. Based on the self-determination theory (Deci and Ryan, 2000), the exclusion theory of anxiety (Baumeister and Tice, 1990), the social compensation model (Kraut et al., 2002), and the reserve capacity model (Gallo and Matthews, 2003), a moderated mediation model was formulated to examine whether peer phubbing would be indirectly related to social networking site addiction *via* increased social anxiety and whether this indirect association was moderated by family financial difficulty. These findings facilitate our understanding of “how” and “for whom” peer phubbing impacts social networking site addiction, and they also provide insights into the intervention of behaviors of social networking site addiction among undergraduates.

### Peer Phubbing and Social Networking Site Addiction

The current study showed that peer phubbing was positively associated with social networking site addiction as our expectation (Hypothesis 1). Although prior studies have identified parental phubbing as a potential influencing factor among children and adolescents being addicted to mobile phones (Hong et al., 2019; Liu et al., 2019; Xie et al., 2019), few studies have explored the relationship between peer phubbing and Internet addiction of undergraduates. Given that peers play a significant role in the well-being of undergraduates (Basson and Rothmann, 2018) and that social exclusion can be positively associated with Internet addiction (Poon, 2018) as well as attachment to social media (David and Roberts, 2017), our finding elucidated the relationship between peer phubbing and social networking site addiction among undergraduates, which is in line with the theoretical expectation of the self-determination theory (Deci and Ryan, 2000). It suggests that being phubbed by peers can be positively linked to excessive Internet use, apart from being phubbed by parents as revealed in earlier studies (Hong et al., 2019; Liu et al., 2019; Xie et al., 2019). This finding is among the first to test the relationship between peer phubbing and social networking site addiction among undergraduates.

### The Mediating Role of Social Anxiety

Consistent with our expectation (Hypothesis 2), social anxiety played a mediating role in the relationship between peer phubbing and social networking site addiction, which extends the earlier theory as well as empirical research. Specifically, it advances our understanding of the exclusion theory of anxiety by applying it into the use of mobile phones during interpersonal interactions. In addition, this study tests the relationship between peer phubbing and social networking site addiction with a probable answer why it happens. Few empirical studies have tested this relationship or examined the mechanism underlying this linkage.

In addition to the overall mediating effect, every independent link of the mediating process is noteworthy. For the link between peer phubbing and social anxiety, the result supported the notion that peer phubbing, a kind of social exclusion (Xie et al., 2019), can induce anxiety (Davidson et al., 2019). Establishing caring interpersonal relationships is one of the main challenges for the youth (Gerrig, 2012). They might experience social anxiety when noticing mobile phones drawing more attention of the peers in their company (Baumeister and Tice, 1990). This finding highlights the importance of supportive atmosphere in school settings as well as good manners during interpersonal interactions among students, which can help to prevent a series of negative consequences.

Regarding the relationship between social anxiety and social networking site addiction, the results indicated that social anxiety was positively related to social networking site addiction, which is consistent with prior studies (Chen et al., 2019; Lin et al., 2019). Individuals who are socially anxious can keep anonymous, and they have more control over the activities during the interaction (Stritzke et al., 2004; Tian, 2013). Besides, online socialization helps alleviate the concern of signs for nervousness that can be physically observed (Darcin et al., 2016). As a result, the less stressful online setting makes it a more secure and comfortable socialization option for individuals who are socially anxious (Kraut et al., 2002; Valkenburg and Peter, 2009). Although it satisfies the socialization need for socially anxious individual, social networking site addiction can be a by-product (Kardefelt-Winther, 2014). This finding is also in line with the theoretical expectation of the social compensation model (Kraut et al., 2002).

### The Moderating Role of Family Financial Difficulty

This study also explored the moderating effects of family financial difficulty on the associations between peer phubbing and social networking site addiction. Family financial difficulty was not only positively related to social anxiety and social networking site addiction but also moderated the association between peer phubbing and social anxiety. Specially, the association between peer phubbing and social anxiety was stronger for undergraduates in higher family financial difficulty. These findings are congruent with the theoretical deduction from the reserve capacity model, which posits that the poor might be more vulnerable for negative social interactions (Gallo and Matthews, 2003). The individuals in high family financial difficulty not only are exposed to more situations requiring usage of their resources but also live in environments preventing the development and replenishment of resources (Gallo and Matthews, 2003). As a result, they are more vigilant (Kraus et al., 2011) and reactive (Kraus et al., 2012) to social threats such as peer phubbing.

### Limitations and Future Directions

The current study has provided a theory on how peer phubbing leads to social networking site addiction. However, several limitations still exist and need to be addressed in future studies. First, the present study considers only the acts of the phubber and the economic background of the phubbee, but the situational and cognitive factors may also play a role. Second, more effort is needed to fully uncover the mechanism between peer phubbing and social networking site addiction. Although the exclusion theory of anxiety (Baumeister and Tice, 1990) suggests that peer phubbing can lead to social anxiety and the social compensation model (Kraut et al., 2002) implies that social networking site addiction might be the further result, there may be other linkages connecting peer phubbing and social networking site addiction. Third, it is unclear whether the findings of our study that are based on convenience sample of college students can be generalized to clinical samples. We were also unclear about the generalizability of current findings to more general outcomes (e.g., Internet addiction). Fourth, this study adopted a cross-sectional design; future research should take a longitudinal or experimental approach to further confirm the causal connections.

### Practical Implications

In spite of the above-mentioned limitations, some practical implications can be obtained from our findings. First, given that peer phubbing is positively associated with the social networking site addiction among undergraduates, a friendly, supportive, and polite atmosphere at school settings should be encouraged. Communication manners should be trained and reminded. The educator should also serve as a role model for students with proper conducts during social interactions. Second, we demonstrated the mediating role of social anxiety in the relationship between peer phubbing and social networking site addiction. In other words, social anxiety is a proximal factor for social networking site addiction. A decreasing social anxiety may also be a possible avenue for interventions of social networking site addiction. Courses about recognizing maladaptive cognitions during social interactions and skills for effective interpersonal communication should be taught, which may help to boost self-confidence as well as to reduce social anxiety. Third, our study found that family financial difficulty played a moderating role in the indirect relationship between peer phubbing and social networking site addiction, which suggests that undergraduates from low-income families should be the focus of intervention, given their high tendency for the development of social anxiety while experiencing peer phubbing.

## Conclusion

In summary, this moderated mediation model is among the first examining the relationship between peer phubbing and social networking site addiction, as well as testing the mediating role of social anxiety and the moderating role of family financial difficulty. We found that peer phubbing was positively linked to social networking site addiction, where social anxiety served as a potential mediation mechanism. Moreover, the first stage of the mediation mechanism was moderated by family financial difficulty. The association between peer phubbing and social anxiety was stronger for undergraduates in higher family financial difficulty.

## Data Availability Statement

The raw data supporting the conclusions of this article will be made available by the authors, without undue reservation.

## Ethics Statement

The studies involving human participants were reviewed and approved by Human Research Committee of School of Economics and Management, Beijing University of Posts and Telecommunications. Written informed consent from the participants' legal guardian/next of kin was not required to participate in this study in accordance with the national legislation and the institutional requirements.

## Author Contributions

XC, SJ, and LL contributed to conception and design of the study. XC, JY, and YC contributed to data collection. XC and XW performed the statistical analysis. XC and SJ wrote the first draft of the manuscript. All authors contributed to manuscript revision, read, and approved the submitted version.

## Conflict of Interest

The authors declare that the research was conducted in the absence of any commercial or financial relationships that could be construed as a potential conflict of interest.

## Publisher's Note

All claims expressed in this article are solely those of the authors and do not necessarily represent those of their affiliated organizations, or those of the publisher, the editors and the reviewers. Any product that may be evaluated in this article, or claim that may be made by its manufacturer, is not guaranteed or endorsed by the publisher.
